# Recovery from Post-Traumatic Amnesia During Inpatient Rehabilitation: A Retrospective Cohort Study

**DOI:** 10.3390/life16020221

**Published:** 2026-01-28

**Authors:** Tay Kai Wen Elvina, Lim Gek Hsiang, Chua Karen

**Affiliations:** 1 Department of Post-Acute & Continuing Care, Jurong Community Hospital, Singapore 609606, Singapore; 2 Yong Loo Lin School of Medicine, National University of Singapore, Singapore 117597, Singapore; karen.chua@nhghealth.com.sg; 3 Health Services Research Unit, Singapore General Hospital, Singapore 169608, Singapore; lim.gek.hsiang@sgh.com.sgl; 4 Institute of Rehabilitation Excellence (IREx), Tan Tock Seng Hospital Rehabilitation Centre, Singapore 307382, Singapore; 5 Lee Kong Chian School of Medicine, Nanyang Technological University, Singapore 639798, Singapore

**Keywords:** traumatic brain injury, post-traumatic amnesia, functional independence measure, rehabilitation

## Abstract

Background: Traumatic brain injury (TBI) is a global healthcare problem, and post-traumatic amnesia (PTA) is a known predictor of long-term and societal outcomes. However, factors influencing PTA recovery during the inpatient rehabilitation phase remain underexplored, particularly in Asian populations. Objective: To identify factors associated with PTA duration and emergence during inpatient rehabilitation and examine their impact on functional outcomes. Materials and Methods: We conducted a retrospective, single-center cohort study over a 7-year period among patients with acute TBI who were admitted to an inpatient rehabilitation hospital. Outcomes included PTA emergence and duration, discharge Functional Independence Measure (FIM), rehabilitation length of stay, and Glasgow Outcome Scale (GOS) at ≥1 year. Results: A total of 100 patients were analyzed. In an adjusted Cox regression, age ≥ 55 years (Hazard Ratio [HR] 0.47) and non-infective medical complications during rehabilitation (HR 0.31) were associated with reduced likelihood of PTA emergence, while mild admission GCS (13–15; HR 4.80) and epidural hemorrhage (EDH) (HR 2.00) were associated with PTA emergence. PTA non-emergence was associated with approximately a 20-point lower discharge FIM total score (adjusted model, *p* < 0.001). A PTA duration of ≥90 days was associated with a lower total discharge FIM score by approximately 45 points compared with those with a PTA duration of <28 days (*p* < 0.001). PTA emergence was associated with better GOS at ≥1 year (odds ratio [OR] 3.92, *p* = 0.02). Conclusion: Both acute injury characteristics and intra-rehabilitation factors were associated with PTA recovery functional outcomes. PTA emergence, beyond PTA duration, was strongly associated with discharge functional status and long-term global outcome, supporting the clinical value of PTA in prognostication, rehabilitation planning, and goal setting.

## 1. Introduction

Traumatic brain injury (TBI) is a major public healthcare problem worldwide and is a significant cause of morbidity and mortality in young and old adults [1,2]. Many individuals with TBI sustain significant cognitive and physical disabilities, affecting their reintegration into the community and work [3]. Post-traumatic amnesia (PTA) is frequently observed among hospitalized patients after TBI and occurs in a significant proportion of individuals with moderate-to-severe injuries [4].

PTA is a clinical entity used to describe a state of disorientation post-TBI and comprises a state of altered consciousness with agitation, amnesia, and confusion [4,5]. The most distinctive feature of PTA is defective memory, which can range from an isolated auditory-recall deficit to a more global memory impairment [5,6,7], in combination with impaired information processing, executive dysfunction, and in some cases, confusion and agitation. Memory impairment following TBI is reported to reflect injury in brain networks supporting learning and memory, including the medial temporal lobe, diencephalic structures, and frontal systems [8].

The PTA duration correlates well with TBI severity [5] and helps with the prognostication of late functional outcomes in TBI patients. Prognostication of functional outcomes after TBI provides valuable information for healthcare providers, facilitating communication with patients and their families and enabling better allocation of resources.

Walker et al. [9] conducted a cohort study of 1332 patients who were enrolled in the National Institute on Disability and Rehabilitation-funded TBI Models systems. The study concluded that a longer PTA duration was associated with poorer functional outcomes based on the 1-year and 2-year Glasgow Outcome Scale (GOS) [9]. In particular, patients who emerged from PTA within 4 weeks had a less than 15% chance of severe disability at 1 year [9]. Conversely, patients with a PTA duration of more than 8 weeks had a less than 10% chance of good recovery in 1 year [9].

A cohort study involving 342 individuals with moderate–severe TBI by Quach et al. concluded that TBI individuals with extremely severe PTA durations of more than 28 days had a significantly greater burden of care, based on their Functional Independence Measure (FIM) [10] scores at 5 years post-TBI [11]. Another study found that the odds of returning to productivity at 1 year significantly decrease by 14% with every additional week of PTA duration [12]. A cohort study by Aldossary et al. concluded that PTA duration is a strong predictor of long-term functional outcome, neurocognitive prognosis, and return to employment [13].

Only a few studies have studied the factors associated with patients’ emergence from PTA, implying gaps in our understanding of PTA recovery. Lindsey et al. studied predictors of recovery from PTA and found that lesion location, especially parietal lobe lesions, and initial Galveston Orientation and Amnesia Test (GOAT) scores were helpful in prognosticating recovery from PTA [14]. However, the study involved only 40 patients and studied mainly non-modifiable factors, such as brain lesion location, age, sex, and cause of injury, and their association with PTA recovery.

Therefore, the objectives of this study were as follows:

Primary objective: To identify factors associated with PTA emergence and PTA duration during inpatient rehabilitation in adults with TBI.

Secondary objective: To examine the association between PTA measures (duration and emergence) and rehabilitation outcomes, including FIM [10], rehabilitation length of stay (LOS), and GOS at ≥1 year.

## 2. Materials and Methods

### 2.1. Study Design and Setting

This retrospective cohort study was conducted at the Tan Tock Seng (TTSH) Hospital Rehabilitation Centre, Singapore. The TTSH Rehabilitation Centre is a 95-bed tertiary rehabilitation center with direct links to a level-1 trauma hospital. The electronic medical records of consecutive TBI admissions to a tertiary rehabilitation center from 1 January 2010 to 31 December 2017 were retrieved and entered into a standardized data collection form.

A research data form was created by extracting only study-relevant variables from the hospital electronic system while excluding all direct identifiers such as names, identification numbers, dates of birth, and addresses. Each record was assigned a unique study code, and the de-identified database is used for analysis without access to identifiable information.

This manuscript was prepared using the Strengthening the Reporting of Observational Studies in Epidemiology (STROBE) [15] guidelines for cohort studies. The complete STROBE checklist is in the Supplementary Material Section (Table S1).

### 2.2. Ethics Statement

The study obtained institutional ethical approval from the National Healthcare Group-NHG Domain Specific Review Boards (DSRB reference number: 2017/01092). It also obtained a waiver of consent as only de-identified data was collected and it was unfeasible to ask for consent from patients who had been discharged and lost to follow-up. All research was conducted in compliance with the WHO Declaration of Helsinki.

### 2.3. Study Population

The inclusion criteria consisted of patients with their first-ever TBI, aged ≥ 18 years, diagnosed by admitting neurosurgeons, confirmed by CT brain imaging, admitted to inpatient rehabilitation at the TTSH rehabilitation center within 90 days of TBI, and those who completed the inpatient rehabilitation program.

Exclusion criteria included those with previous TBI, missing FIM [10] or Westmead Post-Traumatic Amnesia (WPTAS) [6] data, and those in whom the WPTAS could not be accurately administered due to the presence of severe agitation, attention deficits, or communication impairments. Table S2 in the Supplementary Section summarizes the inclusion and exclusion criteria for this study.

### 2.4. Variables for Analysis

Demographic, injury-related, radiological, and clinical variables, such as age, gender, ethnic group, cause of TBI, Glasgow Coma Scale (GCS) on admission, hospital LOS, presence of medical complications (infective and non-infective) during inpatient rehabilitation stay, surgical procedures, and initial radiological findings (admission day CT brain reports), were collected. For this study, infective medical complications were defined as clinically diagnosed infections documented in medical records that required treatment (e.g., urinary tract infection, pneumonia). Non-infective complications were defined as non-infectious medical events that required clinical intervention or monitoring (e.g., seizures, postural hypotension, anemia, or deep vein thrombosis). These variables were extracted retrospectively from inpatient medical documentation and coded as binary variables (present or absent).

Radiological variables were extracted from the initial CT brain report at the time of acute admission and coded as present or absent. Radiological variables included contusion, epidural hemorrhage, subarachnoid hemorrhage, diffuse axonal injury, subdural hemorrhage, mass effect, skull vault fractures, and ventricular compression. Diffuse axonal injury was coded only when stated in CT reports or neurosurgical documentation; however, CT imaging may underestimate diffuse axonal injury relative to MRI, and therefore, misclassification remains possible.

Functional outcomes included rehabilitation LOS, admission and discharge FIM [10] scores, discharge destination, and Glasgow Outcome Scale (GOS) [16] at ≥1 year post injury. GOS [16] at ≥1 year was obtained retrospectively from outpatient records when follow-up documentation was available.

The WPTAS [6] was used to assess PTA presence and emergence and PTA duration in this study. The WPTAS [6] consists of 7 orientation items and 5 memory items that assess prospective memory. The scale is administered on a daily basis, and a patient is said to have emerged from PTA once he or she is able to achieve three consecutive days with a score of 12/12. The WPTAS was administered daily to patients upon admission to inpatient rehabilitation until they were able to achieve three consecutive days of perfect 12/12 scoring or until the day of discharge from inpatient rehabilitation. For this study, the PTA duration was calculated from the TBI date until the date of the first three consecutive 12/12 scores on WPTAS, denoting PTA emergence, or rehab discharge if not emerged. Missing daily WPTAS occurred occasionally due to clinical constraints (e.g., reduced arousal or inability to participate); these were treated as missing data rather than assumed recovery. PTA duration included both pre-rehabilitation days and inpatient rehabilitation days.

Functional outcomes in the study were assessed using FIM [10] and GOS [16]. The Functional Independence Measure (FIM) [10] consists of 18 items in all, including 13 motor items assessing self-care, sphincter control, transfers, and locomotion domains and 5 cognitive items assessing communication and social cognition. Each item is scored from 1 (Total Assistance) to 7 (Complete Independence). The FIM [10] assessment gives a total score from 18 to 126, with higher scores indicating better functional outcomes. Admission and discharge FIM [10] assessments were routinely administered to all patients at the study rehabilitation center by trained healthcare professionals. Admission and discharge FIM [10]s were assessed for all patients within 72 h of admission and 72 h of discharge from rehabilitation center, respectively (Table 1).

WPTAS and FIM assessments were performed by training healthcare professionals as part of routine care. However, a formal interrater reliability assessment was not available for this retrospective study and is acknowledged as a limitation.

The 5-level GOS is used to measure the degree of functional recovery and social participation in the community post-TBI and is the most commonly cited scale in brain injury studies [17]. It comprises 5 categories (I–V) ranging from death (I) to persistent vegetative state (II), severe disability (III), moderate disability (IV), and good recovery (V). GOS scores were reviewed retrospectively from outpatient clinical records at ≥1 year post-TBI.

### 2.5. Statistical Analyses

Univariate analyses were used to describe the demographic and clinical characteristics of the patients, in totality and by the presence or absence of PTA emergence. Numbers and percentages were reported for categorical data such as contusion and ethnic group. Continuous data were first checked to see if they were normally distributed. For normally distributed data, mean and standard deviation were reported; and for skewed data, median and interquartile range were reported to describe their central tendencies and data spread. Continuous variables were summarized using median and interquartile range (IQR) due to non-normal distributions.

Age was dichotomized at ≥55 years old to reflect clinically meaningful older-adult risk stratification used in TBI outcome research. This was based on clinical relevance and prior literature [18,19].

Patients who emerged and did not emerge from PTA were also compared by their demographic and clinical patient characteristics. Associations between PTA emergence and categorical variables were examined using Pearson’s Chi-square test of association if more than 80% of the cells had expected counts of at least 5. Otherwise, Fisher’s exact test was used. Student’s *t*-test was used to perform the comparison for normally distributed data, and the Mann–Whitney test was used to compare skewed data, to examine if and which characteristics differed between patients who emerged and did not emerge from PTA. The level of significance was *p* < 0.05 for all tests.

Variables included in adjusted models were selected a priori based on clinical relevance and established evidence in neurorehabilitation literature, rather than through automated stepwise selection procedures, to reduce risks of overfitting and spurious associations in modest sample.

A Cox proportional hazards regression was used to assess the effect of risk factors (crude and adjusted) on PTA emergence. Patients who emerged from PTA were defined as “events” and patients who did not emerge from PTA were considered to be censored at the end of the study period. A test of the proportionality assumption was also performed to assess the adequacy of the model. The proportional hazards assumption was assessed using standard diagnostic methods, and no major violations were identified. Results are presented with effect estimates and 95% confidence intervals (CI).

The effect of associated factors on late PTA emergence was also examined. If the time-to-emergence was >28 days, it was considered a “late PTA emergence”; and if a patient emerged ≤ 28 days from the first day of the TBI accident, it was considered an “early PTA emergence”. The cut-off of 28 days was chosen as PTA duration of >28 days had been classified as “very severe PTA” in studies and under the Traumatic Brain Injury Model System of Care [10,20]. In this analysis, competing risk analyses were used, whereby a late PTA emergence was defined as an event, and an early PTA emergence was defined as a competing event. Patients who did not emerge from PTA were censored at the end of the study period. This approach allowed identification of factors specifically associated with delayed emergence rather than early recovery.

A binary logistic regression was used to assess the effect of PTA duration on GOS outcomes (≥1 year post discharge) and discharge destination. Linear regression was used to assess the effect of PTA duration on FIM [10] scores at discharge. As the PTA duration was skewed in distribution, it has been categorized into <28 days, 28–89 days, and ≥90 days in the regression modeling. GOS was re-categorized into 2 categories of “Death/PVS/Severe disability” or “Moderate disability/Good recovery”.

Similarly to when examining the association between PTA duration on GOS outcomes and discharge destination, a binary logistic regression was used to assess the effect of PTA emergence on GOS and discharge placement.

The extent of missing data was assessed for each variable and reported in the Supplementary Materials, Table S3. Given the retrospective design and sample size, regression analyses were performed using complete-case analysis. Missing data was not imputed.

All analyses were conducted using STATA 17. (StataCorp. 2021. Stata Statistical Software: Release 17.1. College Station, TX: StataCorp LP).

## 3. Results

### 3.1. Study Cohort and Baseline Characteristics, Stratified by PTA Emergence Status

**Figure 1:**
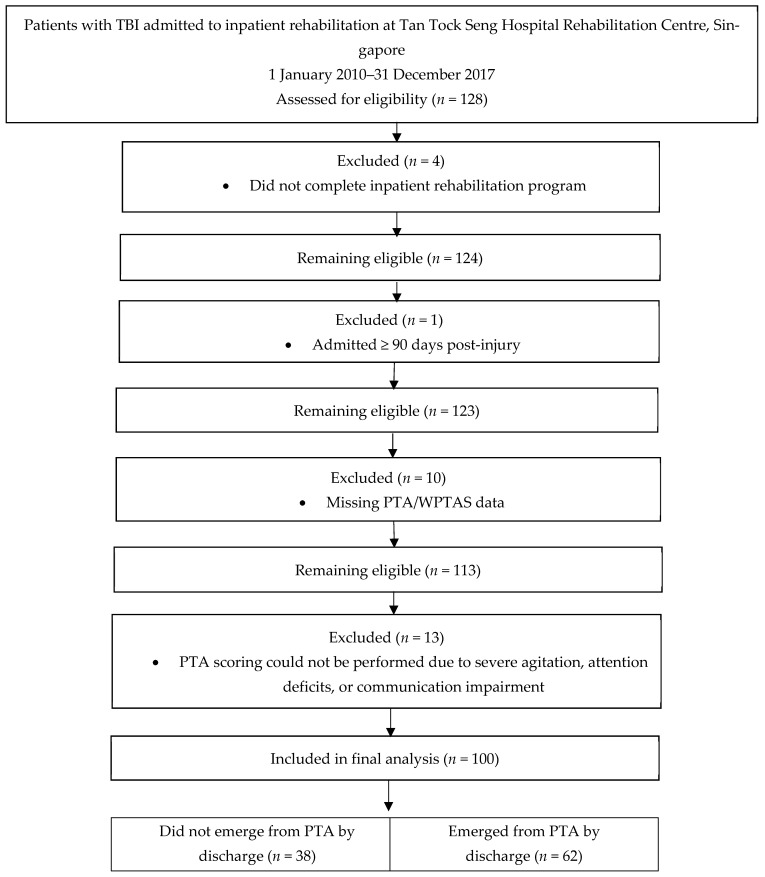
Study flow diagram showing participant selection, exclusions, and final cohort included for analysis.

Of the 128 patients screened during the study period, 28 were excluded due to non-completion of inpatient rehabilitation (*n* = 4), admission of ≥90 days post-injury (*n* = 1), missing PTA data (*n* = 10), or inability to perform PTA scoring (*n* = 13). A participant flow chart showing screening, exclusion, and inclusion is provided (Figure 1).

The baseline characteristics are shown in Table 1. In total, 100 patients met the inclusion criteria and were included in the final analysis.

Table 1 presents the demographic and clinical profiles of the patients, stratified by their PTA emergence status. Overall, 77% (77) of the patients were Chinese, 76% (76) were male, and the median age of the cohort was 60.5 years. Falls were the most common mechanism of injury at 57% (57). At discharge from inpatient rehabilitation, 62% (62) emerged from PTA. Further details of the rehabilitation course, including surgical procedures, blood markers, and medication used, are shown in Supplementary Table S4.

### 3.2. Factors Associated with PTA Emergence

Table 2 presents both the unadjusted and adjusted effects of the potential risk factors on PTA emergence. In the adjusted model, mild admission GCS (13–15), presence of epidural hemorrhage (EDH), younger age (<55), and absence of non-infective complications were independently associated with PTA emergence. Subdural hemorrhage (SDH), diffuse axonal injury (DAI), and infective complications were not significant after adjustment. Conversely, patients aged ≥ 55 years were approximately half as likely to emerge from PTA (HR 0.47; 95% CI: 0.2, 0.9), as compared to patients below 55 years old. As compared to patients with severe GCS, patients with moderate GCS at admission were 2.6 times (95% CI: 1.1, 6.0) as likely to emerge from PTA, and patients with mild GCS at admission were 4.8 times (95% CI: 2.0, 11.4) as likely to emerge from PTA. Patients with EDH were approximately twice as likely to emerge from PTA (HR 2.00) as compared to patients without EDH. Patients with non-infective medical complications were approximately 69% less likely to emerge from PTA (HR 0.31).

### 3.3. Factors Associated with Late PTA Emergence

Competing-risks regression analyses examining factors associated with late PTA emergence (>28 days) are shown in Supplementary Table S5. No variables reached statistical significance in the adjusted model. However, it was noted that the patients with cerebral contusions were 3.5 times as likely as patients without contusions to emerge late from PTA (*p* = 0.07).

### 3.4. Association Between PTA Duration and Functional Outcomes

Longer PTA duration was significantly associated with poorer functional outcomes (Table 3a). Compared with patients who emerged from PTA < 28 days, those with PTA durations of 28–89 days had lower discharge FIM total scores (−13.44 points; 95% CI −22.81 to −4.06), while patients with PTA duration ≥ 90 days demonstrated poorer discharge function (−44.92 points; 95% CI −57.44 to −32.39). The odds of achieving a discharge FIM total score > 90 were significantly lower for patients with a PTA duration of 28–89 days (OR 0.24; 95% CI 0.06–0.90) or ≥90 days (OR 0.04; 95% CI 0.01–0.18), compared with those with a PTA duration < 28 days.

PTA duration ≥ 90 days was associated with reduced rehabilitation efficiency (−0.99; 95% CI −1.41 to −0.57) and longer rehabilitation LOS (+64.54 days; 95% CI 46.82–82.26).

### 3.5. Association Between PTA Emergence and Functional Outcomes

Table 3b shows that patients who emerged from PTA were more likely to have a better outcome, as described by GOS. The odds of patients having moderate disability/good recovery at 1 year were 3.9 times (95% CI: 1.3, 11.8) as likely among patients who emerged as compared to patients who did not emerge. In unadjusted analyses, PTA emergence was associated with a 24.7-point higher discharge FIM [10], which remained significant after adjustment (20.8 points). The odds of having > 90 as the total discharge FIM [10] score among patients who emerged were 4.45 times as much as those of those who did not emerge. A total discharge FIM [10] score of more than 90 corresponds to a score of 5 or more, signifying either modified- or full-independence status (supervision) in each FIM [10] category. PTA emergence was not significantly associated with discharge destination. (OR 0.39, *p* = 0.31)

### 3.6. Factors Associated with FIM [8] Discharge

**Figure 2:**
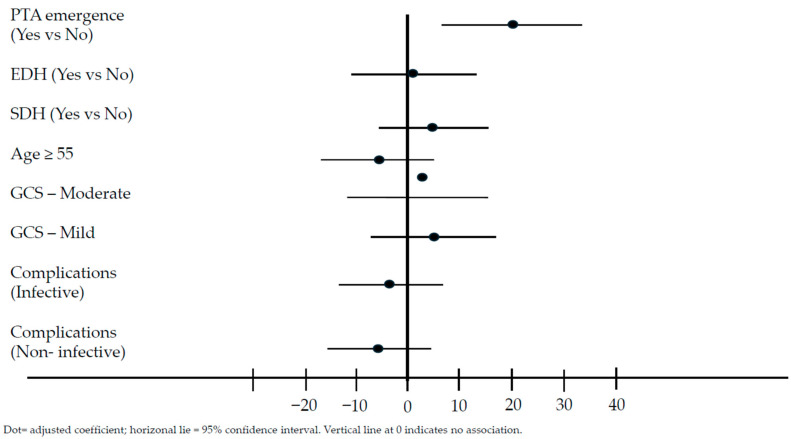
Forest plot of adjusted linear regression coefficients and 95% confidence intervals for factors associated with discharge FIM total score. The vertical line represents the null value (coefficient = 0). Abbreviations: CI, confidence interval; EDH, epidural hemorrhage; FIM, Functional Independence Measure; GCS, Glasgow Coma Scale; PTA, post-traumatic amnesia; SDH, subdural hemorrhage.

Table 4 presents both the unadjusted and adjusted effects of the potential risk factors on FIM [10] discharge. In the adjusted model, it was observed that PTA emergence was significantly associated with higher discharge FIM [10] scores by approximately 20 points compared to non-emerged patients. (*p* = 0.001). Figure 2 provides a forest plot to visually summarize the adjusted regression model for discharge FIM total score.

## 4. Discussion

### 4.1. Study Population and Cohort Characteristics

There are very few Asian TBI studies looking into factors and functional impacts associated with PTA emergence, which may inform local rehabilitation professionals and families regarding future care and prognosis. To our knowledge, this is one of the first Asian studies to examine factors affecting both PTA emergence and duration during inpatient rehabilitation.

This retrospective cohort study examined factors associated with PTA outcomes of emergence and duration and their relationship with functional outcomes in an older Asian population (median age 60 y, and 77% were of Chinese ethnicity). The characteristics of this study’s cohort are similar to those of previous TBI studies evaluating patients in the inpatient neurorehabilitation setting. In this study, 76% of patients were male, similar to data from Traumatic Brain Injury Model Systems (TBIMS) [20]. However, the median age of this study population of 60 years was much older, compared to an average age of 41.87 from the TBIMS data [20]. Chinese ethnic groups formed approximately three-quarters of the study cohort, consistent with the local population distribution of a Chinese majority.

The majority of patients (68%) included in this study had very severe TBI based on PTA duration (>28 days); the median PTA duration was 38 days, and 62% emerged by rehabilitation discharge (Table 1). Gurin et al. (2016) found that the average length of PTA was 38 days in the acute neurorehabilitation setting [14], and a third of patients had not emerged from PTA at the time of rehabilitation discharge, a figure not dissimilar to ours— 38% were non-emerged. Divita et al., in 2017, also found that approximately 24% of patients were still in PTA on discharge from the inpatient rehabilitation unit [21].

### 4.2. Key Finding—Predictors of PTA Emergence and Clinical Implications

In this study, age, admission GCS, and medical complications excluding infective complications were found to be independent predictors of PTA emergence. Initial radiological findings of EDH were found to be independent predictors of PTA emergence.

Not surprisingly, admission GCS, an important early marker of TBI severity, was found to be significantly associated with PTA emergence in this study. Similarly, a cohort study by Sherer et al. in 2008 compared various indices of TBI and found that a decrement of PTA duration of 8.13 days occurred when GCS increased from 4 to 10 [22]. Although the reliability of admission GCS as a severity marker has been questioned in some studies [22,23,24], it is the most common clinical measure of TBI severity [23,25].

One finding of this study was that older adults (≥55 years) were half as likely to emerge from PTA compared to their younger counterparts. Fraser et al. studied the effect of age on cognitive recovery after TBI and found that older TBI patients had a poorer degree of cognitive recovery in terms of processing speed, executive function, and verbal memory [26]. The aging brain has been associated with white matter changes [27,28], gray matter volume atrophy [29], and the reduction in the quality of hippocampal whole-brain structural associations [27], even in healthy individuals. These anatomical changes, together with the aging brain’s postulated reduced ability for neuroplasticity post-TBI [30], could possibly explain the prolonged duration and severity of cognitive sequelae post-TBI in older patients. The functional impact of TBI may be amplified in older patients, underscoring the need for targeted rehabilitation strategies and proactive medical management in the older population.

The relationship between radiological findings and patient outcomes has been studied in numerous studies, including the study of the Marshall Classification and Rotterdam prognostic scale [31,32,33]. Our data showed that patients with EDH on initial radiological findings were more likely to emerge from PTA. EDH commonly results from injury to the middle meningeal artery during head injury. This finding of favorable outcomes of patients with EDH is in accordance with findings from other studies [34,35,36]. An observational study by Gutowski et al. found that isolated EDH was associated with favorable outcomes and low mortality [35], in concordance with our findings, where most patients with EDH had early surgical intervention, in accordance with established brain trauma protocols; this likely contributed to more favorable outcomes. Interestingly, the presence of DAI on initial radiological findings showed an unadjusted association with a higher likelihood of PTA emergence but did not remain significant after adjustment. In contrast, other studies have found the presence of DAI to be associated with poorer functional outcomes, longer PTA duration, and greater cognitive impairment [13,37].

Medical complications were also associated with PTA emergence. Whyte et al. conducted a prospective observational cohort study in 2013 on patients with conscious disorders and found that endocrinal–metabolic medical complications were independent predictors of mortality, and epilepsy predicted poor long-term outcomes [38]. However, this paper by Whyte et al. did not specifically model PTA emergence [38]. To date, no studies have specifically examined the association between medical complications and PTA emergence in adults with TBI. Including medical complications as analytic variables in this study highlights potentially modifiable factors on PTA recovery and reflects the importance of early identification of medical complications and proactive medical management during inpatient rehabilitation.

### 4.3. Functional Implications of PTA Duration and Emergence

This study also examined the relationship between PTA duration and PTA emergence on GOS and FIM [10] outcomes. Longer PTA duration and PTA emergence were both associated with discharge FIM [10] scores, with longer PTA duration associated with lower discharge FIM, while only PTA emergence was associated with GOS outcomes. This finding has been supported in numerous studies. Kosch et al. found that PTA duration alone predicted 30–40% of discharge FIM [10] variance and predicted 24% of variance on GOS at 6 months [39]. Divita et al. found that patients who were still in PTA at discharge had significantly lower FIM [10] scores and lower GOS scores compared to those who had emerged from PTA [21]. Consistent with the international literature, the study findings demonstrate that both PTA duration and emergence status were strongly associated with functional outcomes at discharge. However, in this study, the nature and magnitude of PTA duration and emergence associations differed in clinically meaningful ways. Longer PTA duration showed a clear dose–response relationship with poorer functional independence, with patients who remained in PTA for ≥90 days exhibiting markedly lower FIM [10] Motor, Cognitive, and Total scores at discharge and reduced odds of achieving an FIM [10] Total > 90. Nevertheless, PTA emergence appeared to be an even more powerful predictor of functional outcome. Patients who emerged had substantially higher discharge FIM [10] total scores, were more than four times as likely to reach an FIM [10] Total > 90, and demonstrated greater rehabilitation efficiency and shorter LOS. Emergence was also associated with better global outcomes at ≥1 year. Taken together, these results suggest that while prolonged PTA duration reflects greater injury severity and is associated with diminished functional potential, the point of emergence itself represents a critical cognitive threshold that more directly influences rehabilitation engagement and discharge independence. Thus, PTA emergence demonstrated a strong and independent association with discharge outcomes.

While Dahdah et al. [40] identified PTA duration as a key predictor of discharge functional outcomes, including FIM, our findings suggest that PTA emergence was found to be the only significant variable associated with FIM [10] discharge outcome, after adjustment of other factors such as radiological findings, admission GCS, and medical complications. This study finding supports the importance of routine PTA assessment post-TBI and emphasizes the potential use of this information to help with the estimation and prediction of discharge outcomes.

In addition, PTA emergence was associated with a significantly shorter rehabilitation LOS by approximately 26 days. These findings are consistent with the prior literature. Kosch et al. reported a strong relationship between LOS in the brain injury unit and PTA duration [39]. This information may be useful in the setting of resource allocation and the establishment of rehabilitation goals and LOS by the interdisciplinary team. Notably, PTA non-emergence was not associated with institutionalization in this cohort, which may reflect both the Asian context of strong family support, availability of local paid live-in caregiver hires, and local practice patterns at the TTSH Rehabilitation Centre, where patients who have not yet emerged from PTA may still be discharged home with caregiver support rather than be institutionalized. This contrasts with findings by Kosch et al., who reported that patients who did not emerge from PTA in 6 months post-TBI were more frequently discharged to places other than home [39].

### 4.4. Study Limitations

We highlight the following limitations: firstly, this study had a retrospective design, a small sample size, and data involved patients who were admitted to the rehabilitation unit over a 7-year period ending approximately 8 years ago, during which there could have been program variations and pre-selection bias by admitting rehabilitation physicians and uncertain relevance to current clinical practice. The retrospective study design is subject to information bias due to reliance on routinely documented clinical records rather than prospective research data. This is especially relevant for longer-term outcomes such as GOS at ≥1 year. As GOS follow-up assessments were obtained retrospectively from outpatient records when follow-up documentation was available, follow-up completeness varied across patients, and this may introduce information and attrition biases.

Secondly, missing data were also present for selected variables; therefore, bias may have been introduced if the missing data was not completely at random. Thirdly, the exclusion of patients who were not able to complete inpatient rehabilitation or who could not undergo WPTAS assessment may have systematically excluded certain individuals with more severe neurological deficits. We acknowledge that this may have introduced selection bias and survivor bias and may limit the generalizability of our findings to the broader TBI population.

Fourthly, although adjusted regression models were used, residual confounding remains possible, including confounding by indication. For example, patients with EDH may have had timely neurosurgical intervention, which may partially explain better outcomes. Lastly, given the study’s modest sample size, findings should be interpreted as hypothesis-generating, and further validation in larger cohorts will be required.

In terms of generalizability, the study cohort consisted mainly of older adults with a majority being of Chinese ethnicity treated in Singapore, and therefore, the findings may not fully generalize to younger cohorts or cohorts with different ethnic compositions.

## 5. Conclusions

In conclusion, this study’s findings underscore the critical role of PTA recovery in shaping functional outcomes following TBI. Both the PTA duration and emergence status were strongly associated with discharge functional independence and rehabilitation length of stay, reinforcing PTA emergence as one of the most clinically meaningful indicators of recovery trajectory. These results highlight the importance of routine, standardized PTA measurement within inpatient rehabilitation, where accurate tracking of recovery can directly inform discharge functional prognostication and guide therapeutic planning.

In addition, admission GCS, age, the presence of EDH, and medical complications emerged as relevant contributors to PTA outcomes, suggesting that these variables may enrich future predictive models for stratification of rehabilitation outcomes (FIM) and need for care.

## Figures and Tables

**Table 1:** Baseline demographic and clinical characteristics of cohort, stratified by PTA emergence status (*n* = 100).

Characteristics	Patients Who Did Not Emerge (*n* = 38)	Patients Who Emerged (*n* = 62)	Overall (*n* = 100)	*p*-Value
	No. (%)	No. (%)	No. (%)	
Demographics				
Age: Median (IQR)	66.5 (48–74)	57.5 (34–70)	60.5 (38.5–70)	0.06
Chinese Ethnicity	31 (81.6)	46 (74.2)	77 (77.0)	0.47
Male Sex	30 (78.9)	46 (74.2)	76 (76.0)	0.59
Injury Characteristics				
Cause of TBI				0.60
RTA	17 (44.7)	23 (37.1)	40 (40.0)	
Fall	20 (52.6)	37 (59.7)	57 (57.0)	
Assault	1 (2.6)	0 (0.0)	1 (1.0)	
Sports	0 (0.0)	1 (1.6)	1 (1.0)	
Others	0 (0.0)	1 (1.6)	1 (1.0)	
Admission GCS				0.03
3–8	19 (50.0)	15 (24.2)	34 (34.0)	
9–12	7 (18.4)	14 (22.6)	21 (21.0)	
13–15	12 (31.6)	33 (53.2)	45 (45.0)	
Spinal Injury	5 (13.2)	11 (17.7)	16 (16.0)	0.54
Long Bone Fractures	7 (18.4)	11 (17.7)	18 (18.0)	0.93
Visceral Injuries	8 (21.1)	5 (8.1)	13 (13.0)	0.06
Admission CT findings				
Contusion †	27 (73.0)	44 (71.0)	71 (71.7)	0.83
Subdural Hemorrhage †	32 (86.5)	40 (64.5)	72 (72.7)	0.02
Epidural Hemorrhage †	4 (10.8)	17 (28.3)	21 (21.6)	0.04
Diffuse Axonal Injury †	1 (2.8)	12 (20.0)	13 (13.5)	0.02
Subarachnoid Hemorrhage †	25 (67.6)	43 (70.5)	68 (69.4)	0.76
Mass Effect †	28 (80.0)	38 (62.3)	66 (68.8)	0.07
Ventricular Compression †	13 (37.1)	18 (30.0)	31 (32.6)	0.47
Base of Skull Fracture †	6 (17.1)	10 (17.5)	16 (17.4)	0.99
Closed Skull Vault	15 (44.1)	28 (49.1)	43 (47.3)	0.64
Open Skull Vault †	0 (0.0)	2 (3.5)	2 (2.2)	0.27
Rehabilitation Measures and LOS				
FIM [10] at Admission: Median (IQR)	49 (27–79)	83 (60–97)	75 (47–92)	<0.001
FIM [10] at Discharge: Median (IQR)	90.5 (65–105)	111 (100–120)	106 (87–115)	<0.001
FIM [10] Category at Discharge: >90	19 (50.0)	49 (81.7)	68 (69.4)	0.001
FIM [10] Gain: Median (IQR)	27 (17–42)	26 (15.5–38.5)	26 (17–39)	0.63
FIM [10] Efficiency: Median (IQR)	0.69 (0.40–1.26)	1.20 (0.69–1.77)	0.91 (0.59–1.62)	0.004
PTA Duration, days: Median (IQR)	84 (49–109)	26.5 (18–42)	38 (23.5–77)	<0.001
Rehabilitation LOS, days: Median (IQR)	46 (23–67)	22 (14–36)	26.5 (16–47)	0.0002
Discharge Placement: Home	35 (92.1)	60 (96.8)	95 (95.0)	0.37
Rehabilitation Complications				
Medical Complications (Infection)	26 (68.4)	29 (46.8)	55 (55.0)	0.04
Medical Complications (Others) †	31 (83.8)	30 (48.4)	61 (61.6)	<0.001

Data are presented as median (IQR) or *n* (%) unless otherwise stated. † Variables with missing data. Abbreviations: FIM, Functional Independence Measure; GCS, Glasgow Coma Scale; IQR, Interquartile Range; LOS, length of stay; PTA, post-traumatic amnesia; RTA, road traffic accident; TBI, traumatic brain injury.

**Table 2:** Crude and adjusted Cox proportional hazards models for factors associated with emergence from PTA.

	Crude			Adjusted		
Characteristics	HR	95% CI	*p*-Value	HR	95% CI	*p*-Value
Age						
<55 years	1 (ref)			1 (ref)		
≥55 years	0.64	0.38, 1.07	0.09	0.47	0.22, 0.97	0.04
GCS at Admission			0.009			0.002
Severe GCS 3–8	1 (ref)			1 (ref)		
Moderate GCS 9–12	1.90	0.90, 3.99	0.09	2.61	1.14, 6.00	0.02
Mild GCS 13–15	2.58	1.38, 4.83	0.003	4.80	2.02, 11.40	<0.001
SDH						
No	1 (ref)			1 (ref)		
Yes	0.47	0.27, 0.80	0.006	0.59	0.30, 1.15	0.12
EDH						
No	1 (ref)					
Yes	1.96	1.11, 3.48	0.02	2.00	1.06, 3.76	0.03
DAI						
No	1 (ref)					
Yes	1.94	1.02, 3.70	0.04	0.87	0.34, 2.21	0.76
Medical Complications (Infection)						
No	1 (ref)			1 (ref)		
Yes	0.48	0.28, 0.80	0.005	1.08	0.54, 2.15	0.83
Medical Complications (Non-Infective)						
No	1 (ref)			1 (ref)		
Yes	0.29	0.17, 0.50	<0.001	0.31	0.15, 0.63	0.001
Contusion						
No	1 (ref)			1 (ref)		
Yes	0.78	0.43, 1.40	0.40	0.71	0.36, 1.38	0.31
Neurostimulants						
No	1 (ref)			1 (ref)		
Yes	0.48	0.28, 0.83	0.009	0.70	0.39, 1.26	0.24

Abbreviations: CI, confidence interval; DAI, diffuse axonal injury; EDH, epidural hemorrhage; FIM, Functional Independence Measure; GCS, Glasgow Coma Scale; HR, hazard ratio; PTA, post-traumatic amnesia; SDH, subdural hemorrhage.

**Table 3:** (**a**) Association between PTA duration and functional outcomes at discharge and rehabilitation LOS. Reference group: PTA emergence = No; (**b**) Association of PTA emergence with GOS, discharge function, rehabilitation LOS, and discharge destination.

**(a)**			
**PTA Duration Category**	FIM [10] (Motor) at Discharge (*p* < 0.001)		
	Coefficient	95% CI	*p*-Value
<28 days (ref)	1	-	-
28–89 days	−8.57	−16.09, −1.04	0.03
≥90 days	−31.11	−41.16, −21.06	<0.001
	FIM [10] (Cognitive) at Discharge (*p* < 0.001)		
	Coefficient	95% CI	*p*-value
<28 days (ref)	1	-	-
28–89 days	−4.87	−7.54, −2.20	<0.001
≥90 days	−13.81	−17.37, −10.24	<0.001
	FIM [10] (Total) at Discharge (*p* < 0.001)		
	Coefficient	95% CI	*p*-value
<28 days (ref)	1	-	-
28–89 days	−13.44	−22.81, −4.06	0.005
≥90 days	−44.92	−57.44, −32.39	<0.001
	FIM [10] (Total) at Discharge (Odds of discharge FIM Total >90) (*p* < 0.001)		
	OR	95% CI	*p*-value
<28 days (ref)	1	-	-
28–89 days	0.24	0.06, 0.90	0.03
≥90 days	0.04	0.01, 0.18	<0.001
	FIM [10] (Efficiency) (*p* < 0.001)		
	Coefficient	95% CI	*p*-value
<28 days (ref)	1	-	-
28–89 days	−0.22	−0.54, 0.09	0.17
≥90 days	−0.99	−1.41, −0.57	<0.001
	FIM [10] Gain (Motor) (*p = 0.05*)		
	Coefficient	95% CI	*p*-value
<28 days	1 (ref)	-	-
28–89 days	7.95	1.52, 14.39	0.02
≥90 days	4.13	−4.39, 12.66	0.34
	FIM [10] Gain (Cognitive) (*p = 0.77*)		
	Coefficient	95% CI	*p*-value
<28 days	1 (ref)	-	-
28–89 days	0.99	−1.75, 3.72	0.48
≥90 days	0.58	−3.05, 4.20	0.75
	FIM [10] Gain (Total) (*p = 0.08*)		
	Coefficient	95% CI	*p*-value
<28 days	1 (ref)	-	-
28–89 days	9.33	1.13, 17.53	0.03
≥90 days	5.10	−5.81, 16.01	0.36
	Rehabilitation LOS (Days) (*p* < 0.001)		
	Coefficient	95% CI	*p*-value
<28 days	1 (ref)	-	-
28–89 days	9.23	−3.98, 22.44	0.17
≥90 days	64.54	46.82, 82.26	<0.001
**(b)**			
Outcome	Measure	95% CI	*p*-value
GOS † at ≥1 year	OR	3.92 (1.31 to 11.78)	0.02
FIM (10) (Total) at Discharge	Coefficient	24.68 (15.62 to 33.62)	<0.001
FIM (10) (Total) at Discharge (≤90 vs. >90)	OR	4.45 (1.79 to 11.09)	0.001
FIM (10) (Efficiency)	Coefficient	0.39 (0.08 to 0.69)	0.01
FIM (10) Gain (Total)	Coefficient	−2.58 (−10.12 to 4.96)	0.50
Rehab LOS	Coefficient	−26.28 (−40.08 to −12.48)	<0.001
Discharge Destination	OR	0.39 (0.06 to 2.44)	0.31

FIM [8] (Total) at discharge (≤90 versus >90) is classified as (a) Referent: “≤90”; (b) Outcome: “>90”. Data are presented as regression coefficient or odds ratio (OR) with 95% confidence interval (CI). Abbreviations: CI, confidence interval; FIM, Functional Independence Measure; LOS, length of stay; OR, odds ratio; PTA, post-traumatic amnesia. GOS is classified as (a) Referent: “Death/PVS/Severe disability”, and (b) Outcome: “Moderate disability/Good recovery”. FIM [8] (Total) at discharge (≤90 versus >90) is classified as (a) Referent: “≤90”; (b) Outcome: “>90”. Discharge destination is classified as: (a) Referent: “Home”; (b) Outcome: “Others”. Estimates represent the association of PTA emergence (Yes vs. No) with each outcome. Data are presented as regression coefficient or odds ratio (OR) with 95% confidence interval (CI). † GOS at ≥1 year was available for 67/100 patients. Abbreviations: CI, confidence interval; FIM, Functional Independence Measure; GOS, Glasgow Outcome Scale; LOS, length of stay; OR, odds ratio; PTA, post-traumatic amnesia.

**Table 4:** Crude and adjusted linear regression models for factors associated with discharge FIM total score.

	Crude			Adjusted		
Characteristics	Coefficient	95% CI	*p*-Value	Coefficient	95% CI	*p*-Value
PTA Emergence						
No	1 (ref)			1 (ref)		
Yes	24.68	15.62, 33.74	<0.001	20.80	9.31, 32.30	0.001
EDH						
No	1 (ref)			1 (ref)		
Yes	7.33	−5.03, 19.70	0.24	0.70	−10.96, 12.35	0.91
SDH						
No	1 (ref)			1 (ref)		
Yes	−3.35	−14.68, 7.98	0.56	4.44	−6.26, 15.14	0.41
Age						
<55 years	1 (ref)			1 (ref)		
≥55 years	−10.98	−20.99, −0.96	0.03	−6.80	−18.00, 4.41	0.23
GCS at Admission			0.36			0.69
Severe GCS 3–8	1 (ref)			1 (ref)		
Moderate GCS 9–12	7.39	−6.39, 21.16	0.29	2.40	−11.34, 16.14	0.73
Mild GCS 13–15	7.80	−3.58, 19.20	0.18	5.35	−7.11, 17.82	0.40
Medical Complications (Infection)						
No	1 (ref)			1 (ref)		
Yes	−11.44	−21.29, −1.59	0.02	−3.45	−13.79, 6.90	0.51
Medical Complications (Non-infective)						
No	1 (ref)			1 (ref)		
Yes	−15.67	−25.60, −5.75	0.002	−6.07	−16.87, 4.74	0.27

Abbreviations: CI, confidence interval; EDH, epidural hemorrhage; FIM, Functional Independence Measure; GCS, Glasgow Coma Scale; PTA, post-traumatic amnesia; SDH, subdural hemorrhage.

## Data Availability

The data is not publicly available due to institutional review board regulations.

## Supplementary Materials

The following supporting information can be downloaded at: https://www.mdpi.com/article/10.3390/life16020221/s1, Table S1: STROBE Statement—checklist of items that should be included in reports of observational studies; Table S2: Inclusion and Exclusion Criteria; Table S3: Extent of missing data by variable (n = 100); Table S4: Rehabilitation course, investigations and medications among patients who emerged vs did not emerge (n = 100); Table S5: Crude and Adjusted Competing-Risks Regression Models for Factors Associated with Late PTA Emergence (>28 Days Post- Injury).
