# Supplementary material for: Recovery from Post-Traumatic Amnesia During Inpatient Rehabilitation: A Retrospective Cohort Study
Source: Life (Basel). 2026 Jan 28;16(2):221. doi: 10.3390/life16020221 (PMC12942532; doi:10.3390/life16020221)
Supplement: Supplementary file 1 [file life-16-00221-s001.zip › life-4080388-supplementary.pdf]

Supplementary Section

Table S1: STROBE Statement—checklist of items that should be included in reports of observational studies.

	Item No.	Recommendation	Relevant text from manuscript (section/Page-Line)
**Title and abstract**	1	(*a*) Indicate the study's design with a commonly used term in the title or the abstract	Abstract/Methods section 2.1 Study design and setting
		(*b*) Provide in the abstract an informative and balanced summary of what was done and what was found	Abstract (Backgrounds, Methods, Results, Conclusion)
**Introduction**			
Background/rationale	2	Explain the scientific background and rationale for the investigation being reported	Introduction (Section 1)
Objectives	3	State specific objectives, including any prespecified hypotheses	Introduction (Section 1: primary and secondary aims)
**Methods**			
Study design	4	Present key elements of study design early in the paper	Methods (2.1)
Setting	5	Describe the setting, locations, and relevant dates, including periods of recruitment, exposure, follow-up, and data collection	Methods (2.1)
Participants	6	(*a*) *Cohort study*—Give the eligibility criteria, and the sources and methods of selection of participants. Describe methods of follow-up<br>*Case-control study*—Give the eligibility criteria, and the sources and methods of case ascertainment and control selection. Give the rationale for the choice of cases and controls<br>*Cross-sectional study*—Give the eligibility criteria, and the sources and methods of selection of participants	Methods (2.3) + Results (Section 3.1)
		(*b*) *Cohort study*—For matched studies, give matching criteria and number of exposed and unexposed<br>*Case-control study*—For matched studies, give matching criteria and the number of controls per case	Not applicable (no matching done)

Variables	7	Clearly define all outcomes, exposures, predictors, potential confounders, and effect modifiers. Give diagnostic criteria, if applicable	Methods (Section 2.4 and 2.5)
Data sources/ measurement	8*	For each variable of interest, give sources of data and details of methods of assessment (measurement). Describe comparability of assessment methods if there is more than one group	Methods (Section 2.4: EMR data, FIM, WPTAS, GOS)
Bias	9	Describe any efforts to address potential sources of bias	Discussion- Limitations
Study size	10	Explain how the study size was arrived at	Methods Section. Retrospective cohort so all eligible cases were included.
Quantitative variables	11	Explain how quantitative variables were handled in the analysis. If applicable, describe which groupings were chosen and why	Methods (Section 2.5)
Statistical methods	12	(*a*) Describe all statistical methods, including those used to control for confounding	Methods (Section 2.5)
		(*b*) Describe any methods used to examine subgroups and interactions	Methods (Section 2.5: interaction/subgroup analyses not performed; covariates selected a priori)
		(*c*) Explain how missing data were addressed	Methods (Section 2.5 Statistical analysis; no imputation); Supplementary Table S4 (missingness summary)
		(*d*) *Cohort study*—If applicable, explain how loss to follow-up was addressed<br>*Case-control study*—If applicable, explain how matching of cases and controls was addressed<br>*Cross-sectional study*—If applicable, describe analytical methods taking account of sampling strategy	Results section (GOS availability statement); Limitations section (attrition bias)
		(*e*) Describe any sensitivity analyses	Not done
Results			
Participants	13*	(a) Report numbers of individuals at each stage of study—e.g. numbers potentially eligible, examined for eligibility, confirmed eligible, included in the study, completing follow-up, and analyzed	Results (Section 3.1) + Flowchart (Figure 1)
		(b) Give reasons for non-participation at each stage	Results (Section 3.1: exclusion reasons)
		(c) Consider use of a flow diagram	Flowchart (Figure 1)
Descriptive data	14*	(a) Give characteristics of study participants (e.g. demographic, clinical, social) and information on exposures and potential confounders	Table 1

		(b) Indicate number of participants with missing data for each variable of interest	Supplementary Table S3 (extent of missingness per variable)
		(c) *Cohort study*—Summarize follow-up time (e.g., average and total amount)	Results: rehabilitation length of stay summary; Results: GOS availability statement, Limitations: follow-up completeness.
Outcome data	15*	*Cohort study*—Report numbers of outcome events or summary measures over time	Results (PTA emergence 6=62; non emerged n=38; Tables 2-4)
		Case-control study—Report numbers in each exposure category, or summary measures of exposure	Not applicable
		Cross-sectional study—Report numbers of outcome events or summary measures	Not applicable
Main results	16	(*a*) Give unadjusted estimates and, if applicable, confounder-adjusted estimates and their precision (e.g., 95% confidence interval). Make clear which confounders were adjusted for and why they were included	Tables 2-4
		(*b*) Report category boundaries when continuous variables were categorized	Methods/Results section. (PTA duration < 28, 28-89, ≥90, age: <55/≥55)
		(*c*) If relevant, consider translating estimates of relative risk into absolute risk for a meaningful time period	Not required (hazard ratios and coefficients used)
Other analyses	17	Report other analyses done—e.g. analyses of subgroups and interactions, and sensitivity analyses	Supplementary Table S1 (late PTA emergence)
Discussion			
Key results	18	Summarize key results with reference to study objectives	Discussion (section 4)
Limitations	19	Discuss limitations of the study, taking into account sources of potential bias or imprecision. Discuss both direction and magnitude of any potential bias	Discussion- limitations
Interpretation	20	Give a cautious overall interpretation of results considering objectives, limitations, multiplicity of analyses, results from similar studies, and other relevant evidence	Discussion (section 4)
Generalizability	21	Discuss the generalizability (external validity) of the study results	Discussion (section 4), Limitations on generalizability/external validity and Singapore context, older and majority Chinese ethnicity cohort.
Other information			
Funding	22	Give the source of funding and the role of the funders for the present study and, if applicable, for the original study on which the present article is based	Funding section. No external funding.

Table S2: Inclusion and Exclusion Criteria

Inclusion:	Exclusion:
• First-ever TBI, age ≥18<br>• CT-confirmed TBI<br>• Admitted within 90 days<br>• Completed inpatient rehab	• Previous TBI<br>• Missing FIM or WPTAS<br>• Severe agitation/attention/communication deficits preventing valid WPTAS administration

Abbreviations: CT, computed tomography; FIM, Functional Independence Measure; TBI, traumatic brain injury; WPTAS, Westmead Post-Traumatic Amnesia Scale.

Table S3: Extent of missing data by variable (n = 100)

Variable	Missing No. (%)
Age	0 (0%)
Ethnicity	0 (0%)
Gender	0 (0%)
Cause of TBI	0 (0%)
Admission GCS	0 (0%)
Spinal Injury	0 (0%)
Long Bone Fractures	0 (0%)
Visceral Injuries	0 (0%)
Radiology: Contusion	1 (1%)
Radiology: Subdural Hemorrhage	1 (1%)
Radiology: Epidural Hemorrhage	3 (3%)
Radiology: Diffuse Axonal Injury	4 (4%)
Radiology: Subarachnoid Hemorrhage	2 (2%)
Radiology: Mass Effect	4 (4%)
Radiology: Ventricular Compression	5 (5%)
Radiology: Base of Skull Fracture	8 (8%)
Radiology: Closed Skull Vault	9 (9%)
Radiology: Open Skull Vault	9 (9%)
FIM [8] at Admission	0 (0%)
FIM [8] at Discharge	0 (0%)
PTA Duration	0 (0%)
Rehabilitation Length of Stay (LOS), days	0 (0%)
Antiplatelet/ Anticoagulant prior to admission	1 (1%)
Medical Complications (Infection)	0 (0%)
Medical Complications (Others)	1 (1%)
Lowest Hemoglobin Level during Admission	0 (0%)
Highest INR during Admission	0 (0%)
Surgical Procedures	0 (0%)
Tracheostomy	0 (0%)
Neurostimulants	1 (1%)
Discharge Placement	0 (0%)
GOS at ≥ 1 year	33 (33%)
PTA emergence status by discharge	0 (0%)
PTA duration (days)	0 (0%)

Table S4: Rehabilitation course, investigations and medications among patients who emerged vs did not emerge (n = 100)

Characteristics	Patients who did not emerge (n=38)	Patients who emerged (n=62)	Overall (n=100)	*p*-value
	No. (%)	No. (%)	No. (%)	
**Rehabilitation course, investigations and medications.**				
**Antiplatelet/ Anticoagulant prior to admission†**	8 (21.6)	12 (19.4)	20 (20.2)	0.79
**Lowest Hemoglobin Level during Admission:** Median (IQR)	9.8 (7.9 – 11.0)	10.7 (8.6 – 11.7)	10.4 (8.5 – 11.5)	0.13
**Highest INR during Admission:** Median (IQR)	1.2 (1.1 – 1.3)	1.15 (1.1 – 1.2)	1.2 (1.1 – 1.3)	0.10
**Surgical Procedures**	27 (71.1)	36 (58.1)	63 (63.0)	0.19
**Tracheostomy**	8 (21.1)	6 (9.7)	14 (14.0)	0.11
**Neurostimulants†**	22 (59.5)	22 (35.5)	44 (44.4)	0.02

Data are presented as median (IQR) or n (%) unless otherwise stated.

† Variables with missing data.

Abbreviations: INR, International Normalized Ratio; IQR, Interquartile Range.

Table S5: Crude and Adjusted Competing-Risks Regression Models for Factors Associated With Late PTA Emergence (>28 Days Post- Injury)

	Crude			Adjusted		
**Characteristics**	**SHR**	**95% CI**	**P-value**	**SHR**	**95% CI**	**P-value**
**Age**						
<55 years	1 (ref)			1 (ref)		
≥55 years	0.97	0.47, 2.02	0.94	1.52	0.56, 4.10	0.41
**GCS at Admission**			0.44			0.81
Severe GCS 3-8	1 (ref)			1 (ref)		
Moderate GCS 9-12	1.74	0.67, 4.51	0.26	1.25	0.40, 3.88	0.70
Mild GCS 13-15	1.60	0.68, 3.74	0.28	1.41	0.47, 4.23	0.54
**SDH**						
No	1 (ref)			1 (ref)		
Yes	0.65	0.31, 1.38	0.26	0.69	0.27, 1.75	0.44
**EDH**						
No	1 (ref)			1 (ref)		
Yes	1.91	0.86, 4.25	0.11	1.76	0.77, 4.02	0.18
**DAI**						
No	1 (ref)			1 (ref)		
Yes	2.36	1.05, 5.30	0.04	2.61	0.70, 9.70	0.15
**Medical Complications (Infection)**						
No	1 (ref)			1 (ref)		
Yes	1.22	0.58, 2.56	0.60	1.14	0.43, 2.98	0.80
**Medical Complications (Others)**						
No	1 (ref)			1 (ref)		
Yes	0.81	0.39, 1.69	0.57	1.08	0.46, 2.52	0.86
**Contusion**						
No	1 (ref)			1 (ref)		
Yes	3.07	0.92, 10.27	0.07	3.51	0.92, 13.34	0.07
**Neurostimulants**						
No	1 (ref)			1 (ref)		
Yes	0.56	0.26, 1.18	0.13	0.48	0.20, 1.17	0.11

Abbreviations: CI, confidence interval; DAI, diffuse axonal injury; EDH, epidural haemorrhage; GCS, Glasgow Coma Scale; SDH, subdural haemorrhage; SHR, subhazard ratio.
